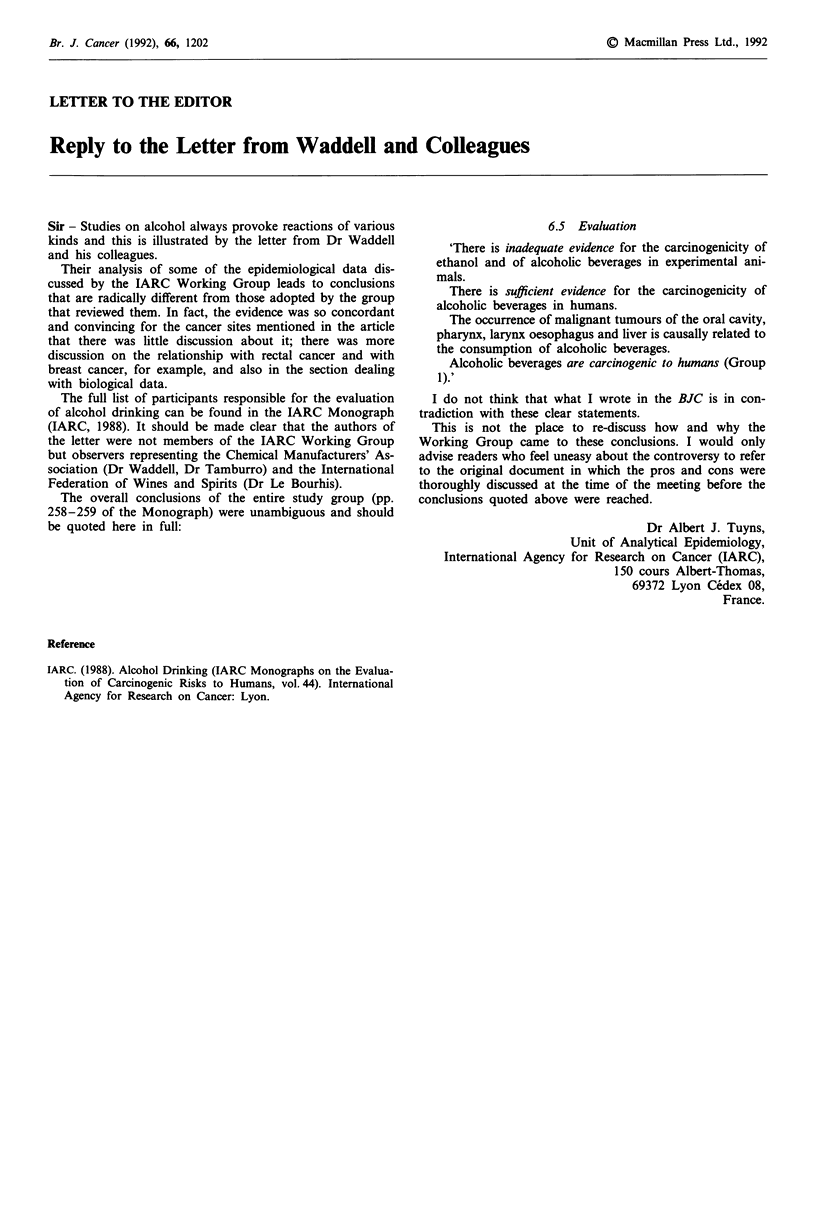# Reply to the Letter from Waddell and Colleagues

**Published:** 1992-12

**Authors:** Albert J. Tuyns


© Macmillan Press Ltd., 1992

### LETTER TO THE EDITOR

### Reply to the Letter from Waddell and Colleagues

**Sir** – Studies on alcohol always provoke reactions of various kinds and this is illustrated by the letter from Dr Waddell and his colleagues.

Their analysis of some of the epidemiological data discussed by the IARC Working Group leads to conclusions that are radically different from those adopted by the group that reviewed them. In fact, the evidence was so concordant and convincing for the cancer sites mentioned in the article that there was little discussion about it; there was more discussion on the relationship with rectal cancer and with breast cancer, for example, and also in the section dealing with biological data.

The full list of participants responsible for the evaluation of alcohol drinking can be found in the IARC Monograph (IARC, 1988). It should be made clear that the authors of the letter were not members of the IARC Working Group but observers representing the Chemical Manufacturers' Association (Dr Waddell, Dr Tamburro) and the International Federation of Wines and Spirits (Dr Le Bourhis).

The overall conclusions of the entire study group (pp. 258–259 of the Monograph) were unambiguous and should be quoted here in full:

*6.5 Evaluation*

'There is *inadequate evidence* for the carcinogenicity of ethanol and of alcoholic beverages in experimental animals.

There is *sufficient evidence* for the carcinogenicity of alcoholic beverages in humans.

The occurrence of malignant tumours of the oral cavity, pharynx, larynx oesophagus and liver is causally related to the consumption of alcoholic beverages.

Alcoholic beverages *are carcinogenic to humans* (Group 1).'

I do not think that what I wrote in the *BJC* is in contradiction with these clear statements.

This is not the place to re-discuss how and why the Working Group came to these conclusions. I would only advise readers who feel uneasy about the controversy to refer to the original document in which the pros and cons were thoroughly discussed at the time of the meeting before the conclusions quoted above were reached.

Dr Albert J. Tuyns,
Unit of Analytical Epidemiology,
International Agency for Research on Cancer (IARC),
150 cours Albert-Thomas,
69372 Lyon Cédex 08,
France.

### Reference

IARC. (1988). Alcohol Drinking (IARC Monographs on the Evaluation of Carcinogenic Risks to Humans, vol. 44). International Agency for Research on Cancer: Lyon.